# Trisomy 19 and T(9;22) In a Patient with Acute Basophilic Leukemia

**DOI:** 10.1155/2011/269491

**Published:** 2011-09-13

**Authors:** Alicia Rojas-Atencio, Karelis Urdaneta, Marisol Soto-Quintana, Francisco Alvarez Nava, Jenny Cañizales, Ernesto Solis

**Affiliations:** Genetic Medical Unity, Faculty of Medicine, University of Zulia, Maracaibo, Venezuela

## Abstract

We report a case of acute basophilic leukemia with two coexisting clonal abnormalities, t(9;22) and trisomy 19. The blast showed positive reaction with myeloperoxidase but negative reaction with chloroacetate esterase and acid phosphatase. Metachromatic features of the blast were observed with toluidine blue stain. Ultrastructure study showed the presence of azurophilic granules in basophils and blast mast cells. Conventional and molecular cytogenetic studies revealed, t(9;22) with *BCR/ABL* positive and trisomy 19 in all metaphase cells. To our knowledge, this paper here is the first to present acute basophilic leukemia with trisomy 19 and t(9;22).

## 1. Introduction

The chromosomal changes in patients with leukemia constitute a fundamental event in the onset, progression and transformation of this disorder. A mechanism of the action has been recognized to some of these chromosomal alterations into of these transformations [1, 2]. Usually, only one primary chromosome change is present in the same leukemic clone [3–7]. But two or more chromosomal anomalies have been described in few reports, especially in basophilic leukemia (BL) [8].

BL is an extremely rare malignant hematological disease which has been described in a few reports accounting for 4-5% of all cases of acute nonlymphocytic leukemia [9, 10]; most cases of basophilic leukemia have been reported to be a consequence of the evolution from other malignant hematological disorders [11]. It was first described as early as in 1906 by Joachin and Uber in two patients with extreme basophilia and clinical features of myelocytic leukemia [12]. This leukemia has recently been included into a revised classification of leukemia by the World Health Organization (WHO) panel [13]. Diagnosis is based on morphological, cytochemical, and ultra structural analyses of basophil and blast cells [14]. Response to treatment is variable in patients with BL and consistent diagnosis criteria are lacking due to the rarity of the disease. 

**Figure 1:**
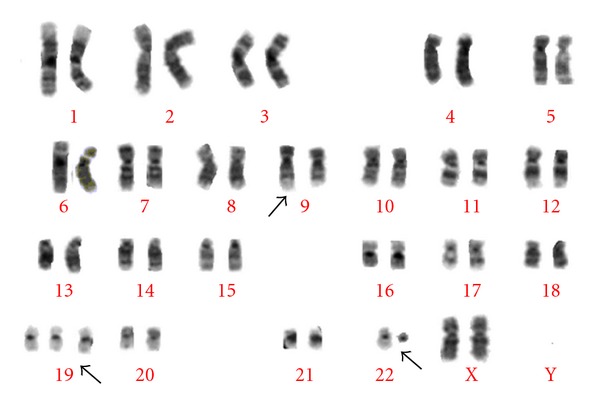
G-Banded karyotype of one of the leukemic clones showing 47, XY, +19,  + t(9;22)(q34;q11); arrows indicate the structurally and numerical abnormal chromosomes.

In this context the objective of our study is to report an additional case of BL with two clonal abnormalities, t(9;22), and trisomy 19 (Figure 1) in all metaphases analyzed.

## 2. Case Report

The patient was a 44-year-old Venezuelan female which was referred to our center with asthenia, fatigue, fever, productive cough and respiratory difficulty. Previous past medical and chuirurgical antecedents were not significant. On examination, the patient had hepatosplenomegaly grade II. Laboratory findings revealed anaemia (Hb 5 g/dL), and hyperleucocytosis (total leukocyte count was 23, 1 × 10^9^/L) with the following differential count: 23% polymorphs, 20% lymphocytes, 9% monocytes, 2% eosinophils, 2% myelomonocytes, 4% metamyelocytes and 40% basophils; (platelets 120 × 10^9^/L). Bone marrow was infiltrated by 80% of cells with basophils granules in their cytoplasm and 20% of immature nuclei. Only a few blast cells were shown to be positive with Myeloperoxidase (MPO), the basophilic blast and the mature basophils were metachromatic in the toluidine blue stain, negative for the chloroacetate esterase, and, no reaction with acid phosphatase, Auer rods were absent. Immunophenotyping showed that the blasts were positive for CD34, CD33, and CD9 and negative for HLA-DR and CD14. Ultrastructural analysis reported the presence of azurophilic granules. Conventional cytogenetic studies using standard culture methods and GTG banding showed a trisomy of the chromosome 19 and translocation 9;22 on the 20 metaphases analyzed (Figure 1). The presence of the BCR/ABL complex was detected by FISH analysis (Vysis, extrasignal) in 95% of the nuclei analyzed. Thus, myeloid chronic leukemia (CML) in blastic crisis was diagnostic. The patient was treated with oral hydroxyurea (2 g) with partial improvement, but she died two weeks later.

## 3. Discussion

Basophilic leukemia has recently been considered as an independent clinical entity in the classification of acute myeloid leukemia by the WHO committee [12].The acute form of this type of leukemia was first described by Wick et al. in 1982 [14] but most of the cases of basophilic leukemia have been reported as an event developed secondary to CML or myelodysplastic syndrome [11]. No specific diagnostic criteria and treatment have been considered due to the heterogeneity of its presentation [9, 10, 14]. Morphologically, basophilic leukemia is also a heterogeneous group where cases with no basophilic differentiation could be seen and blasts may either be agranular or exhibit course basophilic granules in the cytoplasm. Cytochemically, blasts are often negative for myeloperoxidase or nonspecific esterase but they show metachromasia with toluidine blue and diffuse reaction with acid phosphatase. Basophile blast cells express a myeloid phenotype; these are normally positive for CD9 and CD25 which are basoassociated markers [14]. Cytogenetically, basophilic leukemia is not associated with specific chromosomal abnormality. However, cytogenetic studies are necessary in all cases to diagnosis early blast crisis of CML as most of the cases develop secondary to CML. Our patient has distinct morphological differentiation towards basophiles with the presence of immature basophils, its presented positive reaction for MPO. This finding has been described in only two cases [15, 16] and probably indicates the mixed myeloid nature of the blasts with negative reaction for nonspecific esterase and acid phosphatase. These negative reactions indicate the presence of basophils. In the present case, the blasts expressed a myeloid phenotype being positive to the CD33 and CD9 phenotypes which together with the positive for CD34 expression point out to a morphological maturation of the blasts toward the basophils. 

In our patient cytogenetical analyses showed trisomy of the chromosome 19 and presence of the Philadelphia chromosome. To the best of our knowledge, this case may be the first report of basophilic leukemia secondary to CML with trisomy 19. It has been described as the sole chromosomal abnormality in chronic myelomonocytic leukemia, in acute myeloid leukemia and in other myelodysplastic disorders [17, 18]. Chronic myeloid leukemia is the first diagnosis proposed in this patient due to the presence of positive chromosome Philadelphia, however, this patient did not have positive history to CML, and in the other hand, the rapidly fatal progressive evolution as well as positive myeloid markers, and positive MPO, make the diagnosis of acute basophilic leukemia with two coexisting clonal abnormalities (trisomy 19 and t(9;22) chromosome) very likely. However, the Philadelphia chromosome had been described in other haematological malignant diseases [19, 20]. 

We conclude, based on clinical features and fatal violent evolution, that this patient had a form of acute basophilic leukemia with the presence of the positive chromosome Philadelphia and trisomy 19. Due to these results found in this case, a better definition of diagnostic criteria to acute basophilic leukemia is necessary.
